# An Unusual Case of Synchronous Carcinoid of Ovary and Gall Bladder

**DOI:** 10.1155/2013/737016

**Published:** 2013-11-26

**Authors:** Rupali Bassi, Raksha Arora, Sangeeta Bhasin, Nita Khurana

**Affiliations:** ^1^Department of Obstetrics and Gynaecology, Lok Nayak Hospital, Maulana Azad Medical College, New Delhi 110002, India; ^2^Department of Pathology, Lok Nayak Hospital, Maulana Azad Medical College, New Delhi 110068, India

## Abstract

Multifocal carcinoid is a known phenomenon. We present a rare combination of an ovarian carcinoid synchronous with gallbladder carcinoid. This rare combination has not been reported so far. An asymptomatic 45-years-old perimenopausal lady was diagnosed to have a metastatic ovarian cancer, but on laparotomy she was found to have a primary synchronous metastatic gall bladder as well. On histopathological evaluation she was found to have two separate primary carcinoids. Subsequently the patient received chemotherapy and is completely asymptomatic on follow up. Further research needs to be undertaken and guidelines need to be formulated for management of these cases.

## 1. Introduction

Carcinoid tumors are neuroendocrine tumors derived predominantly from the enterochromaffin or Kulchitsky cells [1]. Carcinoid tumors were first described as early as 1888 by Lubarsh, occurring in the small bowel [2, 3]. The overall incidence of carcinoids is estimated to be approximately 1 to 2/100000 individuals [4]. They may be found anywhere in the human body but have been described traditionally as originating from the foregut, midgut, or hindgut.

Multifocal primary disease is rarely known to occur with carcinoids, although very few cases have been reported till date. Synchronous primary carcinoid tumors have been reported in about 0% to 3% of small bowel carcinoid, and it has been observed that a second primary malignancy occurs in 7% to 32% of cases [5]. We present a rare case of synchronous primary carcinoid in the ovary and gall bladder, which has not been reported so far.

## 2. Case

45-year-old para 4, living 4 presented to the outpatient department with chief complaints of a progressive abdominal distension over the last 5 months and amenorrhea since the 5 past 3 months. Apart from these the patient was completely asymptomatic.

On examination the general condition of the patient was stable and abdominal examination revealed a soft cystic, nontender abdominopelvic lump, corresponding to 22-week uterine size. The gall bladder was also palpably enlarged in the right hypochondrium. On Speculum examination the cervix and vagina were healthy, and vaginal examination revealed bilateral nontender cystic masses with restricted mobility.

Ultrasound showed a left ovarian cystic mass in the iliac region with the gall bladder showing a localized heterogeneous echogenic mass. There was minimal dilatation of the common bile duct. This was followed by a contrast enhanced computed tomography scan of the patient that showed a multiseptated mass with solid and cystic components in the adnexal region till the level of L3. The gall bladder fossa showed a heterogeneously enhancing lesion but no lymphadenopathy.

Chest X-ray, upper gastrointestinal endoscopy along with colonoscopy were within normal limits. The CA-125 and CEA levels were 102.7 units/mL and 8.23 ng/mL, respectively. Fine needle aspiration cytology of the mass suggested the features of epithelial neoplasia. During preoperative evaluation the malignancy appeared advanced, and hence it was planned for a staging and debulking surgery as a stage IV malignancy. She underwent an exploratory laparotomy with total abdominal hysterectomy, bilateral salpingo o-ophorectomy, infracolic omentectomy, hepatic resection and, cholecystectomy. The segment IV B/5 along with the gallbladder was removed.

Intraoperatively there was 200 mL of nonhemorrhagic straw colored ascitic fluid. A right-sided ovarian mass of 20 × 15 cms with solid and cystic areas was noted along with a left ovarian mass of 15 × 15 cms. The uterus and bilateral tubes were looking normal. A liver nodule of 5 × 5 cms in segment 5 on cut section of the gall bladder mucosa was intact on naked eye examination, which later on turned out to be infiltrated by histopathological examination.

**Figure 1:**
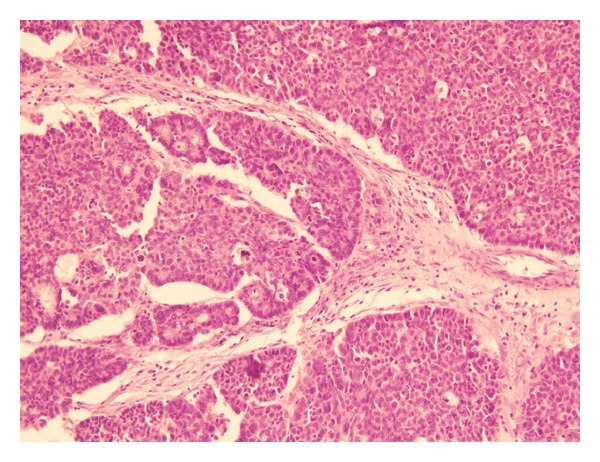
Section from the ovarian tumor showing nests of cells, focally arranged in a trabecular pattern, with prominent nuclear palisading (H and E stain, 10X).

**Figure 2:**
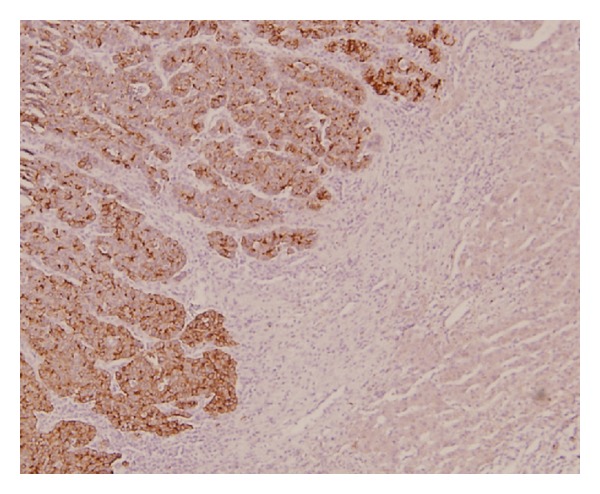
The tumor cells expressing strong immunoreactivity for neuron specific enolase (NSE), while the surrounding hepatocytes are negative.

**Figure 3:**
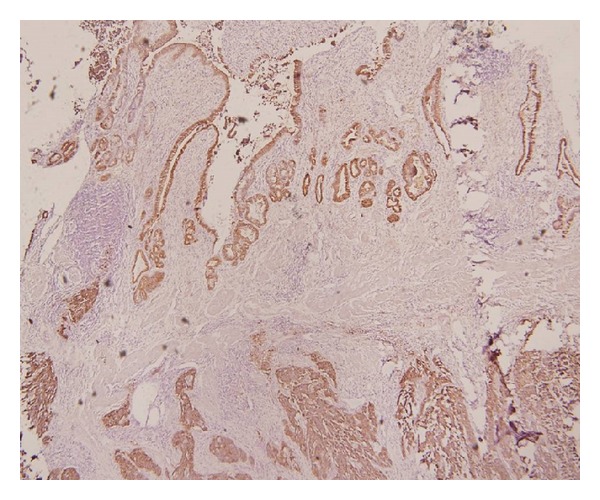
IHC for EMA, staining the normal gall bladder epithelium and the nests of tumor cells infiltrating the wall of the gall bladder.

The peritoneal or the ascitic fluid was free of malignant cells. The right-sided ovarian tumor showed polygonal pleomorphic cells with high mitosis and capsular involvement (Figure 1). Immunohistochemical studies showed expression of epithelial membrane analyzer, chromogranin A, and neuron specific enolase (Figures 2 and 3), thereby suggesting a neuroendocrine carcinoma.

The gall bladder also showed similar features of neuroendocrine tumor which was positive for EMA, chromogranin A, and neuron specific enolase. The tumor was infiltrating the liver, but the margins of the liver nodules were free of the tumor. Interestingly enough the omentum and lymph nodes, as well as the ascitic fluid, were free of any malignant cells.

Patient had an uneventful postoperative period. Two weeks after surgery patient was started on chemotherapy, which was continued for 5 cycles. She received cisplatin (20 mgs), ifosofamide (2 gms) with mesna (400 mgs) and adriamycin (30 mgs). The patient has been asymptomatic during her followup for ongoing chemotherapy.

## 3. Discussion

The incidence of carcinoids is approximately 0.1% of the ovarian neoplasms, whereas amongst the carcinoid clan they are found in 1% of the cases [6].

Carcinoid tumors have a higher incidence in the 5th and the 6th decades of life. The tumor has a predilection for the female gender and is more common in the African American population [7]. Our patient was 45 years old at the time she presented with the symptoms.

Carcinoid tumors of the ovary have been classified under the category of malignant ovarian germ cell tumors. Amongst the malignant germ cell tumors, carcinoids of the ovary are categorized under the monodermal malignant teratomas. Primary ovarian carcinoids are most commonly known to occur unilaterally, in stage I that is not associated with any metastatic disease [8]. Primary gall bladder carcinoids are a rare entity with an incidence of less than 1% of all of the carcinoids [9]. However in our patient there was evidence of a metastatic liver nodule, which could arise from the gall bladder.

Carcinoids are asymptomatic in about 40% to 60% of cases. The typical symptoms of carcinoid syndrome are due to the production and release of serotonin in the systemic circulation. Carcinoid syndrome characterized by facial flushing, diarrhea, bronchospasm, and edema has been observed to occur in about one third of patients with ovarian carcinoid tumors [10]. As with the majority of cases our patient also did not present with any symptoms pertaining to the carcinoid syndrome.

Carcinoid tumors are identified on the basis of the granule-associated proteins chromogranin, synaptophysin, and neuron specific enolase by; mmunohistochemistry [11, 12]. Chromogranin A level is an independent prognostic factor and elevated levels are associated with poor prognosis [13]. In our patient, the immunohistochemical studies done for both the ovarian and the gall bladder malignancies showed a positivity for the epithelial membrane analyzer, chromogranin A, and neuron specific enolase hence classifying them into the neuroendocrine group.

The strategies of management would depend not only on the site, size, and histopathological characteristics of the tumor but also the symptomatology and presence of carcinoid syndrome.

The management of an ovarian carcinoid tumor would range from unilateral salpino-oophorectomy to a total abdominal hysterectomy. Robby et al. in his study on 10 cases had shown complete remission following unilateral oophorectomy [10]. Thus in young patients with unilateral disease, salpingo-oophorectomy is an adequate treatment [14–16], whereas, in cases of aggressive variants of carcinoid as goblet cell carcinoid of appendix where there is an increased risk of metastasis, a prophylactic hysterectomy is advised [16]. In our patient being of the perimenopausal age group a radical procedure was performed.

At present there is no consensus on the role of chemotherapy in cases of ovarian carcinoid. Some authors believe that there is no role of chemotherapy with early stage carcinoid tumors.

There have been a few case reports of patients with gynecological literature regarding successful treatment of advanced ovarian carcinoid following chemotherapy and radiotherapy. In patients with metastatic disease, chemotherapy with 5-fluorouracil (5-FU) and leucovorin is offered [7].

The prognosis in our patients is uncertain as this combination has not so far been studied and the patient had synchronous primary tumors of localized spread to the liver. So far the patient has been asymptomatic after the six months of followup.

## 4. Conclusion

A very rare case of a synchronous combination of ovarian with gall bladder carcinoid has been successfully managed with a multidisciplinary approach; however, long term followup still needs to be done. We need to study these cases more extensively to formulate guidelines for patient management.

## References

[1] Solicia C, Capella C, Buffa R, Johnson LR (1981). Endocrine cells of the digestive system. *Physiology of the Gastrointestinal Tract*.

[2] Ueber LO (1888). den primaren Krebs des Ileum nebst Bemerkungen iiber das gleichzeitige Vorkommen von Krebs und Tuberculos. *Virchows Archives*.

[3] Lu Cortez L, Clemente C, Puig V, Mirada A (1994). Carcinoid tumor: an analysis of 131 cases. *Revista Cl?nica Espa?la*.

[4] Memon MA, Nelson H (1997). Gastrointestinal carcinoid tumors: current management strategies. *Dieases of Colon Rectum*.

[5] Modlin IM, Lye KD, Kidd M (2003). A 5-decade analysis of 13, 715 carcinoid tumors. *Cancer*.

[6] Raut CP, Kulke MH, Glickman JN, Swanson RS, Ashley SW (2006). Carcinoid tumors. *Current Problems in Surgery*.

[7] Robboy SJ, Norris HJ, Scully RE (1975). Insular carcinoid primary in the ovary: a clinicopathologic analysis of 48 cases. *Cancer*.

[8] Zou YP, Li WM, Liu HR, Li N (2010). Primary carcinoid tumor of the gallbladder: a case report and brief review of the literature. *World Journal of Surgical Oncology*.

[9] Robboy SJ, Scully RE, Norris HJ (1977). Primary trabecular carcinoid of the ovary. *Obstetrics and Gynecology*.

[10] Jensen RT, Doherty GM, Hellman VTJ, Rosenberg SA (2001). Carcinoid tumors and the carcinoid syndrome. *DeVita Cancer: Principles and Practice of Oncology*.

[11] Kloppel G, Heitz PU (1994). Classification of normal and neoplastic neuroendocrine cells. *Annals of New York Academy of Sciences*.

[12] Janson ET, Holmberg L, Stridsberg M (1997). Carcinoid tumors: analysis of prognostic factors and survival in 301 patients from a referral center. *Annals of Oncology*.

[13] Davis KP, Hartmann LK, Keeney GL, Shapiro H (1996). Primary ovarian carcinoid tumors. *Gynecolgic Oncology*.

[14] Robboy SJ, Scully RE (1980). Strumal carcinoid of the ovary: an analysis of 50 cases of a distinctive tumor composed of thyroid tissue and carcinoid. *Cancer*.

[15] Brunaud L, Antunes L, Sebbag H, Bresler L, Villemot JP, Boissel P (2001). Ovarian strumal carcinoid tumor responsible for carcinoid heart disease. *European Journal of Obstetrics and Gynecol Reproductive Biology*.

[16] Pahlavan PS, Kanthan R (2005). Goblet cell carcinoid of the appendix. *World Journal of Surgical Oncology*.

